# Exclusive breastfeeding and length of hospital stay in premature infants at a Brazilian reference center for kangaroo mother care

**DOI:** 10.1016/j.jped.2024.01.004

**Published:** 2024-03-21

**Authors:** Patrícia de Padua Andrade Campanha, Maria Clara de Magalhães-Barbosa, Arnaldo Prata-Barbosa, Gustavo Rodrigues-Santos, Antônio José Ledo Alves da Cunha

**Affiliations:** a Universidade Federal do Rio de Janeiro (UFRJ), Faculdade de Medicina, Rio de Janeiro, RJ, Brazil; b Secretaria Municipal de Saúde do Rio de Janeiro - Maternidade Leila Diniz, Rio de Janeiro, RJ, Brazil; c Instituto D'Or de Pesquisa e Ensino (IDOR), Rio de Janeiro, RJ, Brazil; d Universidade Federal do Rio de Janeiro (UFRJ), Instituto de Puericultura e Pediatria Martagão Gesteira, Rio de Janeiro, RJ, Brazil

**Keywords:** Preterm infant, Kangaroo mother-care method, Neonatal intensive care unit, Breastfeeding

## Abstract

**Objective:**

To evaluate exclusive breastfeeding at discharge and hospital length-of-stay in preterm infants undergoing or not the Kangaroo-Mother Care Method (KMC).

**Methods:**

A retrospective cohort study was conducted including preterm infants < 1800 g admitted to the neonatal unit of a KMC reference center. The infants were grouped into the KMC group and the non-KMC group. Multiple logistic and Poisson regressions were performed to evaluate the association between the KMC and two outcomes, exclusive breastfeeding at discharge, and hospital length-of-stay, adjusted for potential confounders.

**Results:**

115 mother-infant dyads were included, 78 in the KMC group and 37 in the non-KMC group. In the bivariate analysis, the KMC group had a lower prevalence of maternal adverse conditions (6% vs. 32%, *p* < 0.001), a higher number of prenatal visits (median 6 vs. 3.5, *p* < 0.001), higher gestational ages (median 32 vs. 31 weeks, *p* < 0.05), higher birth weights (median 1530 vs. 1365 g, *p* < 0.01), a lower prevalence of necrotizing enterocolitis (3.8% vs. 16.2%, *p* < 0.05), parenteral nutrition (50% vs. 73%, *p* < 0.05), and deep vascular access (49.7% vs. 78.4%, *p* < 0.01), a higher prevalence of exclusive breastfeeding (65% vs. 8%, *p* < 0.001) and a shorter length of hospital stay (median 28 vs. 42 days, *p* < 0.001). In the multiple regression analysis, the KMC group was 23 times more likely to be exclusively breastfed at discharge (OR = 23.1; 95% CI = 4,85–109,93) and had a 19% reduction in the hospital length-of-stay (IDR = 0.81; 95% CI = 0.76–0.86) compared to the non-KMC group.

**Conclusions:**

The KMC is associated with better short-term neonatal outcomes and should be encouraged in all Brazilian maternity hospitals.

## Introduction

Every year, an estimated 15 million infants are born prematurely (before 37 completed weeks of gestation). Premature birth is a global problem, ranging from 5% to 18% of live births across countries. Prematurity is the leading cause of death in children under the age of 5 years, responsible for approximately 1 million deaths each year.^1^ In addition, prematurity and its complications are responsible for disability with long-term detrimental effects on motor, cognitive, linguistic, and emotional development. They may cause a substantial emotional and economic cost to the families, as well as an impact on the public health services and other social support systems.^2^^,^^3^ Therefore, effective evidence-based interventions that can be implemented at large scales are urgently needed to reduce the incidence of preterm birth complications and neonatal mortality. In 2022, the WHO published new recommendations for the care of preterm infants, which reflect new evidence that simple and cost-effective interventions can substantially reduce morbidity and mortality in preterm and low birth weight infants. Among them is the Kangaroo-Mother Care Method (KMC).^4^

KMC was conceived in 1979, in Colombia,^5^ and, since 2003, the World Health Organization (WHO) recognized this method as a standard care for low-birth-weight infants, for being a safe, effective, and low-cost care. KMC was defined by WHO as early, continuous, and prolonged skin-to-skin contact between the baby and his mother, exclusive breastfeeding, and early discharge.^6^

Numerous studies have shown that KMC is a safe, effective, and multifaceted intervention with many short-term and long-term positive effects for preterm infants. Scientific evidence has proven KMC benefits when compared to conventional care, such as lower mortality; decreased risk of neonatal sepsis, hypothermia, hypoglycemia, and rehospitalization; increased exclusive breastfeeding rates and mother-infant bonding; decreased physiological responses to pain; better growth, motor, and cognitive development.^7^, ^8^, ^9^, ^10^, ^11^

In Brazil, KMC has been a National Health Policy since 2000 and includes the father's participation, welcoming of the family, individualized care, organization of assistance, and control of the environment. The Ministry of Health and National Reference Centers coordinated several strategies to disseminate the method.^12^, ^13^, ^14^ However, the implementation of KMC in Brazil is still low.^15^

Despite extensive international publications on the benefits of KMC, few Brazilian studies evaluated the association between KMC and neonatal outcomes. This study aims to evaluate the association between the KMC and two outcomes, respectively: exclusive breastfeeding at discharge, and hospital length-of-stay in preterm infants undergoing or not the Kangaroo-Mother Care Method (KMC) in a Brazilian KMC reference center.

## Methods

A retrospective cohort study included preterm infants < 1800 g admitted to the neonatal unit of a public maternity hospital in Rio de Janeiro, a reference center for the KMC, from January to December 2018. KMC, in Brazilian public institutions, has three stages, two in-hospitals and one at home. The first stage is performed in the Neonatal Intensive Care Unit (NICU) and the Conventional Neonatal Intermediate Care Unit where the mother initiates skin-to-skin contact intermittently but does not remain hospitalized with her baby. The second stage is performed in the Kangaroo Neonatal Intermediate Care Unit where the mother remains hospitalized with her baby full-time. In this stage, all care processes initiated in the first stage are maintained, with particular attention to breastfeeding. The third stage is conducted at home and consists of continuing skin-to-skin contact and monitoring the baby until it reaches the weight of 2500 gs.^13^^,^^14^^,^^16^ This study evaluated the in-hospital KMC stages.

All dyads of mother-preterm infants with a birth weight below 1800 g who were admitted to the neonatal unit during the study period were selected. The exclusion criteria were major congenital malformations, genetic syndromes, and infants discharged or who died less than seven days of hospitalization. Data from the participants were extracted from medical records, entered in the REDCAP data bank (Vanderbilt University, Nashville, TN, USA), and exported to Excel spreadsheets (Microsoft Corporation, USA).

The participant dyads were grouped into the KMC group or the non-KMC group. The KMC group included all mother-infant dyads who participated in at least one in-hospital stage of KMC. The non-KMC group consisted of those who did not participate in any stage of the KMC during hospitalization, due to the family's decision/inability, despite all the efforts of the health team. The infants in the non-KMC group were assisted with the same standardized protocols of the neonatal unit but did not have the presence of their parents for skin-to-skin contact.

Perinatal and neonatal variables were described according to the groups. Perinatal variables included maternal age, adverse conditions, underlying diseases, number of prenatal visits, gestational and birth complications, antenatal corticosteroids, and delivery mode. Neonatal variables were gender, twin birth, gestational age, birth weight, resuscitation at the delivery room, Apgar at 5 min, early neonatal complications (respiratory distress syndrome, pneumonia, pulmonary hemorrhage, early sepsis, persistent ductus arteriosus, and shock), late neonatal complications (intraventricular hemorrhage/periventricular leukomalacia, late sepsis, bronchopulmonary dysplasia, necrotizing enterocolitis, and retinopathy of prematurity), treatment (invasive mechanical ventilation, antibiotics, parenteral nutrition, and deep vascular access), length of hospital stay, breastfeeding at discharge and outcome (death or discharge).

Categorical variables were described as proportions, and numerical variables were described as medians and interquartile ranges (IQR). Statistical tests were performed to compare these estimates between the two groups (Mann-Whitney for medians, and Chi-square or Fisher tests for proportions). The outcomes assessed were late neonatal complications, length-of-stay, exclusive breastfeeding at hospital discharge, and mortality. Multiple logistic and Poisson regressions were performed to evaluate the association between the KMC and two neonatal outcomes, respectively – exclusive breastfeeding at discharge and length of hospital stay, accounting for the minimal sufficient adjustment sets of variables identified through directed acyclic graphs (DAGs).^17^ A significance level of 5% was adopted for all analyses, using the statistical program R (R Core Team, Vienna, Austria).

The study was approved by the Research Ethics Committee of the Municipal Health Secretary of Rio de Janeiro under the number 3.593.196.

## Results

Of 223 preterm infants admitted to the neonatal unit during the study period, 139 had birth weights below 1800 g. Of these, 24 infants were excluded due to the following reasons: two had genetic syndromes, 5 had major congenital malformations, and 17 died within less than seven days of life. A total of 115 infants were included in the study, 78 in the KMC group and 37 in a non-KMC group. Among the 78 babies included in the KMC group, 12 participated only in the first stage of the KMC. These 12 infants had lower median gestational age (30.0, IQR 26;31.5) and birth weight (1212 g, IQR 1066;1530 g) and were more severely ill, which limited their participation in the second stage.

In the bivariate analysis, some significant differences between groups were identified. KMC group had a lower prevalence of maternal adverse conditions (6% vs. 32%, *p* < 0.001), a higher number of prenatal visits (median 6 vs. 3.5, *p* < 0.001), higher gestational ages (median 32 vs. 31 weeks, < 0.05), and higher birth weights (median 1530 vs. 1365 g, *p* < 0.01) (Table 1). KMC group had also a lower prevalence of necrotizing enterocolitis (3.8% vs. 16.2%, *p* < 0.05), parenteral nutrition (50% vs. 73%, *p* < 0.05), and deep vascular access (48.7% vs. 78.4%, *p* < 0.01), a higher prevalence of exclusive breastfeeding (65% vs. 8%, *p* < 0.001) and a shorter length of hospital stay (median 28 vs. 42 days, *p* < 0.001). Overall, four neonatal deaths occurred (3.5%), two in the KMC group (2.6%) and two in the non-KMC group (5.4%) with no significant difference (Table 2). The average gestational age and birth weight of the babies who died was 26 weeks and 946 g, respectively. The two KMC group babies died during the first stage of KMC at the NICU.

**Table 1:** Maternal characteristics during pregnancy and neonatal characteristics at birth in KMC group and non-KMC group.

Variables	Total	KMC group	Non-KMC group	p-value*
	*n* = 115	*n* = 78	*n* = 37	
**Perinatal variables**				
Maternal age, years				
Median (IQR)	26 (23; 30.5)	26 (22; 31)	26 (24; 30)	0.549
Adverse conditions^1^, n (%)				
Yes	17 (14.8)	5 (6.4)	12 (32.4)	**< 0.001**
Underlying diseases^2^, n (%)				
Yes	27 (23.5)	21 (26.9)	6 (16.2)	0.303
Prenatal clinic visits				
Median (IQR)	5 (3.5; 7.0)	6 (5; 7.8)	3.5 (0.8; 5.3)	**< 0.001**
Gestational complications^3^, n (%)				
Yes	115 (100)	78 (100)	37 (100)	–
Antenatal corticosteroid, n (%)				
Yes	89 (77.4)	62 (79.5)	27 (73.0)	0.588
Mode of deliver, n (%)				
Vaginal	38 (33.0)	23 (29.5)	15 (40.5)	0.335
Cesarean section	77 (67.0)	55 (70.5)	22 (59.5)	
**Neonatal variables**				
Gender, n (%)				
Male	59 (51.3)	39 (50.0)	20 (54.1)	0.836
Female	56 (48.7)	39 (50.0)	17 (45.9)	
Twin birth, n (%)				
Yes	26 (22.6)	17 (21.8)	9 (24.3)	0.949
Gestational age (GA), weeks				
Median (IQR)	32 (30; 33)	32 (31; 33)	31 (28; 32)	**0.019**
Birthweight (BW), g				
Median (IQR)	1450 (1212; 1658)	1530 (1284; 1692)	1365 (1030; 1555)	**0.004**
BW x GA, n (%)				
AGA	77 (67.0)	50 (64.1)	27 (73.0)	
SGA	38 (33.0)	28 (35.9)	10 (27.0)	0.464
LGA	0 (0.0)	0 (0.0)	0 (0.0)	
Resuscitation at delivery room, n (%)				
Yes	43 (37.4)	25 (32.1)	18 (48.6)	0.131
Apgar score at 5 min				
Median (IQR)	9 (8; 9)	9 (8; 9)	9 (8; 9)	0.081

AGA, appropriate for gestational age; SGA, small for gestational age; LGA, large for gestational age.

1 Adverse condition (violence in childhood, domestic violence, illicit drug use, smoking, alcoholism, homeless).

2 Underlying diseases (chronic hypertension, hypothyroidism, psychiatric disorders, diabetes, asthma, obesity).

3 Gestational complications (hypertension, diabetes, premature labor, premature rupture of membranes, urinary tract infection, chorioamnionitis, congenital infection, pre-eclampsia, eclampsia, fetal centralization, acute fetal distress, placental abruption, chorioamnionitis, maternal sepsis, prolonged amniorrhexis, HELLP syndrome (acronym *Hemolysis, Elevated Liver enzymes, Low Platelet* count).

⁎ p-values (chi-square or Fisher test for categorical variables and Mann-Whitney test for continuous variables).

**Table 2:** Neonatal morbidities and outcomes in the KMC group and non-KMC group.

Neonatal outcomes	Total	KMC group	Non-KMC	p-value*
	*n* = 115	*n* = 78	*n* = 37	
Early neonatal complications, n (%)	78 (67.8)	52 (66.7)	26 (70.3)	0.863
RDS	51 (44.3)	35 (44.9)	16 (43.2)	1.000
Pneumonia	28 (24.3)	16 (20.5)	12 (32.4)	0.247
Pulmonary hemorrhage	7 (6.1)	3 (3.8)	4 (10.8)	0.209
Early sepsis	59 (51.3)	35 (44.9)	24 (64.9)	0.071
PDA	16 (13.9)	8 (10.3)	8 (21.6)	0.175
Shock	10 (8.7)	5 (6.4)	5 (13.5)	0.288
Late neonatal complications, n (%)	30 (26.1)	16 (20.5)	14 (37.8)	0.080
IVH/Periventricular Leukomalacia	13 (11.3)	6 (7.7)	7 (18.9)	0.112
Late sepsis	24 (20.9)	13 (16.7)	11 (29.7)	0.172
Bronchopulmonary dysplasia	15 (13.0)	8 (10.3)	7 (18.9)	0.239
NEC	9 (7.8)	3 (3.8)	6 (16.2)	**0.030**
Retinopathy of prematurity	5 (4.3)	2 (2.6)	3 (8.1)	0.326
Treatment				
IMV, n (%)	36 (31.3)	20 (25.6)	16 (43.2)	0.092
IMV, days, median (IQR)	10.5 (4.0; 24.8)	6.5 (2.8; 28.0)	15.5 (5.0; 22.5)	0.435
Antibiotics, n (%)	66 (57.4)	40 (51.3)	26 (70.3)	0.085
Antibiotics, n schemes, median (IQR)	1.0 (1.0; 2.0)	1.0 (1.0; 2.0)	1.0 (1.0; 2.0)	0.501
Parenteral nutrition, n (%)	66 (57.4)	39 (50.0)	27 (73.0)	**0.034**
Parenteral nutrition, days	6.0 (5.0; 10.8)	5.0 (4.0; 9.5)	7.0 (5.0; 14.5)	0.067
Deep vascular access, n (%)	67 (58.3)	38 (48.7)	29 (78.4)	**0.005**
Deep vascular access (days), median (IQR)	10.0 (7.0; 18.5)	10.0 (5.5; 17.0)	14.0 (8.0; 22.0)	0.185
Exclusive breastfeeding at discharge, n (%)				
Yes	54 (47.0)	51(65.4)	3 (8.1)	
No	57 (49.6)	25 (32.1)	32 (86.5)	**< 0.001**
NA	4 (3.5)	2 (2.6)	2 (5.4)	
Length of hospital stay, days				
Median (IQR)	31.0 (21.5; 47.5)	28.0 (18.3; 37.0)	42.0 (31.0; 61.0)	**< 0.001**
Hospital outcome, n (%)				
Discharged	111 (96.5)	76 (97.4)	35 (94.6)	0.593
Death	4 (3.5)	2 (2.6)	2 (5.4)	

IMV, invasive mechanical ventilation; IQR, Interquartile range; NA, Not available; RDS, respiratory distress. syndrome; PDA, persistent ductus arteriosus; IVH, intraventricular hemorrhage; NEC, necrotizing enterocolitis.

⁎ p-values (chi-square or Fisher test for categorical variables and Mann-Whitney test for continuous variables).

The minimal sufficient set of adjustment variables identified in the DAG for estimating the total effect of KMC on breastfeeding included prenatal visits, maternal age, maternal adverse conditions, maternal underlying diseases, gestational age, birth weight, early and late neonatal complications, invasive ventilation, deep vascular access, parenteral nutrition. The adjustment variables for estimating the effect of KMC on the length of hospital stay were gestational age, birth weight, early and late neonatal complications, invasive ventilation, parenteral nutrition, and deep vascular access (Figure 1 A and B in the supplementary material).

In the multiple regression analysis, the chance of exclusive breastfeeding at discharge was 23 times higher in the KMC group compared to the non-KMC group (OR 23.1; 95% CI 4,85,109,93), while the presence of late neonatal complications reduced the chance of this outcome by 88% (OR 0.12; 95% CI 0.02, 0.61) (Table 3). The hospital length of stay remained 19% shorter (Incidence Density Rate 0.81; 95% CI 0.76, 0.86) in the KMC group, while it was 20% higher in infants with early neonatal complications (OR 1.2; 95% CI 1.09, 1.31), 27% higher in infants with late neonatal complications (OR 1.27; 95% CI 1.17, 1.39), 15% higher in infants submitted to invasive mechanical ventilation (OR 1.15; 95% CI 1.05, 1.26) and 32% higher in infants undergoing parenteral nutrition (OR 1.32; 95% CI 1.16, 1.51) (Table 4).

**Table 3:** Logistic regression results to estimate the effect of Kangaroo Mother Care on exclusive breastfeeding at discharge.

Exclusive breastfeeding at discharge KMC group vs. non-KMC group			
	Crude OR (95% CI)	Adj. OR (95% CI)	P-value
Kangaroo group	21.08 (5.87,75.65)	23.1 (4.85,109.93)	< 0.001
Gestational age (weeks)	1.49 (1.19,1.87)	1.05 (0.69,1.61)	0.806
Prenatal visits (n)	1.12 (0.99,1.26)	0.96 (0.81,1.16)	0.694
Twin birth	0.43 (0.17,1.12)	0.27 (0.07,1.05)	0.057
Maternal adverse conditions^1^	0.19 (0.05,0.73)	0.27 (0.05,1.52)	0.13
Birth weight (g)	1.0029 (1.0013,1.0045)	0.9995 (0.9953,1.0037)	0.81
Early neonatal complications^2^	0.48 (0.22,1.09)	0.86 (0.22,3.31)	0.829
Late neonatal complications^3^	0.12 (0.04,0.39)	0.12 (0.02,0.61)	0.007
Invasive mechanical ventilation	0.23 (0.09,0.58)	0.53 (0.12,2.41)	0.41
Parenteral Nutrition	0.3 (0.14,0.66)	0.5 (0.05,4.82)	0.552
Deep Vascular access	0.25 (0.11,0.56)	1.33 (0.19,9.03)	0.773

Log-likelihood = −46.6417.

No. of observations = 110.

AIC value = 117.2835.

1 Maternal adverse conditions ((violence in childhood, domestic violence, illicit drug use, smoking, alcoholism, homeless).

2 Early neonatal complications (respiratory distress syndrome, pneumonia, pulmonary hemorrhage, early sepsis, persistent ductus arteriosus, shock).

3 Late neonatal complications (intraventricular hemorrhage/periventricular leukomalacia, late sepsis, bronchopulmonary dysplasia, necrotizing enterocolitis, retinopathy of prematurity).

**Table 4:** Poisson regression results to evaluate the effect of Kangaroo Mother Care on the length of hospital stay.

Length of hospital stay (days) KMC group vs. non-KMC group			
	Crude IDR (95% CI)	Adj. IDR (95% CI)	P-value
Kangaroo group	0.64 (0.61,0.68)	0.81 (0.76,0.86)	< 0.001
Gestational age (weeks)	0.8662 (0.857,0.8755)	1 (0.9811,1.0193)	1
Birth weight (g)	0.9985 (0.9984,0.9986)	0.9994 (0.9992,0.9996)	< 0.001
Early neonatal complications^1^	1.83 (1.7,1.97)	1.2 (1.09,1.31)	< 0.001
Late neonatal complications^2^	2.14 (2.01,2.27)	1.27 (1.17,1.39)	< 0.001
Invasive mechanical ventilation	2.17 (2.04,2.3)	1.15 (1.05,1.26)	0.002
Parenteral Nutrition	2.26 (2.11,2.42)	1.32 (1.16,1.51)	< 0.001
Deep Vascular access	2.17 (2.02,2.32)	0.99 (0.87,1.12)	0.86

Log-likelihood = −520.0501.

No. of observations = 115.

AIC value = 1058.1003.

1 Early neonatal complications (respiratory distress syndrome, pneumonia, pulmonary hemorrhage, early sepsis, persistent ductus arteriosus, shock).

2 Late neonatal complications (intraventricular hemorrhage/periventricular leukomalacia, late sepsis, bronchopulmonary dysplasia, necrotizing enterocolitis, retinopathy of prematurity).

## Discussion

This study was conducted in a baby-friendly maternity hospital, a reference center for KMC, where humanized care is already practiced, consisting of individualized care, welcoming the family, encouraging the participation of parents in neonatal care and breastfeeding, environmental control, and pain prevention. As the definitions of KMC practice differ among various countries, and care practices also vary in different units, it is important to highlight the engagement of the family and the health team in the method implemented in this maternity hospital. The parents stay in the neonatal unit and the entire health team encourages breastfeeding and their participation in infant care. Participation in the method significantly increased the proportion of exclusive breastfeeding and decreased the hospital length of stay.

Evidence about the influence of KMC on better neonatal outcomes is largely available worldwide, and the findings of this study corroborate the literature data. In a systematic review and meta-analysis that included 21 studies and enrolled 3042 infants, Conde-Agudelo and Diaz Rosello (2016) demonstrated that KMC was associated with an increased likelihood of exclusive breastfeeding at discharge compared to conventional neonatal care (66.3% vs 56.3%; RR 1.16, 95% CI 1.07 to 1.25.^8^ In another systematic review and meta-analysis that included 124 studies, Boundy et al. also demonstrated that KMC was associated with increased exclusive breastfeeding compared to conventional care (RR 1.50; 95% CI 1.26, 1.78).^7^ Length of hospital stay did not differ significantly between KMC and control groups in both meta-analyses. In a more recent systematic review and meta-analysis that included twelve international studies and 816 infants, Narcisoet al. demonstrated a reduction in the length of hospital stay in days in the KMC group compared to the conventional care group (RR −1.75, 95% CI −3.22 to −0.28).^18^

Despite the wide international literature, there is little Brazilian scientific production evaluating KMC's association with the neonatal outcomes evaluated in this study. Lamy Filho et al. conducted a prospective study including 985 infants with birth weight < 1750 g from 16 Brazilian neonatal units, eight reference centers for the Kangaroo Mother Care, and eight conventional neonatal units. The authors demonstrated a higher rate of exclusive breastfeeding at discharge in the infants from the KMC units, with a relative risk of 2,34 (95% CI 1,13 to 4,82). There was no significant difference in the length of hospital stay.^19^ Almeida et al. conducted a prospective observational study in low-birth-weight infants < 2000 g, comparing the periods before and after the implementation of Kangaroo Mother Care. Rates of exclusive breastfeeding at discharge were higher in the 23 infants in the post-KMC period compared with the 20 infants in the pre-KMC period (82.6% vs. 0%; *p* = 0.00). No differences in birth weight, gestational age, and Apgar score were observed between groups.^20^

Exclusive breastfeeding is associated with short and long-term benefits related to immunological, nutritional, and neurodevelopmental aspects, being the ideal nutrition for preterm and low birth weight newborns.^21^^,^^22^ Therefore, higher rates of exclusive breastfeeding at hospital discharge directly impact the morbidity and mortality of this vulnerable population. This study demonstrated an important impact of KMC on the rate of exclusive breastfeeding at hospital discharge, despite the opposite effect of the presence of late neonatal complications on this outcome.

The reduction in hospital length-of-stay can significantly contribute to the reduction in hospital costs,^23^ in addition to lower neonatal risks related to prolonged hospitalization, improving short- and long-term prognosis. In this study, the KMC group maintained a nineteen percent reduction in the hospital length of stay, despite the opposite effect of several variables that prolonged this outcome such as early and late neonatal complications, invasive mechanical ventilation, and parenteral nutrition. This outcome may reflect the occurrence of clinical complications during hospitalization. A lower prevalence of necrotizing enterocolitis was observed in the KMC group, a complication related to short and long-term morbidity and mortality. No significant differences were observed in other late neonatal complications.

This study has some limitations. This is a single-center study in a reference maternity hospital for KMC, which guarantees that the groups received the same standardized care and minimizes the influence of different care practices on outcomes when comparing different services. On the other hand, due to the incorporation of humanized care and the encouragement of participation in KMC by the entire hospital team, the non-KMC group was reduced. In addition, the sample size was determined by the study period, but despite no sample size calculation positive associations were observed between KMC and the outcomes studied, although, with low precision in some findings. Moreover, several maternal, and neonatal factors associated with non-adherence to KMC, may also influence neonatal outcomes as confounding factors. As assessed in a previous study in a slightly larger sample of this same maternity hospital,^24^ conditions associated with full adherence to the two KMC stages compared to non-adherence to any stage were high school education, presence of a partner, no maternal adverse conditions, and no neonatal resuscitation. The small group of mothers who participated only in the first stage had the most premature and sickest infants. In the present study, the non-KMC group compared to the KMC group (one or two stages) had a higher prevalence of adverse maternal conditions, lower frequency of prenatal visits, lower birth weight, and gestational age, and higher frequency of some late neonatal complications and invasive treatments. These differences could influence the rates of breastfeeding and hospital length of stay. Therefore, all these variables were included in the multiple regression models, but the effect of KMC on the studied outcomes remained positive even after adjustment for them. Still, due to the retrospective design, some essential neonatal variables, probably associated with neonatal outcomes, could not be assessed. Some of these variables were the infant's severity score, the infant's age in days at the beginning of the KMC, and the length of stay in the kangaroo position. Future prospective studies should consider all these aspects for a more comprehensive analysis. Finally, this study was limited to the in-hospital stages of KMC and did not evaluate the third home stage after hospital discharge and long-term outcomes.

In conclusion, this study corroborates the positive effect of KMC, implemented as recommended by the Brazilian Ministry of Health, in increasing exclusive breastfeeding and reducing the length of hospital stay. KMC should be encouraged in all Brazilian maternity hospitals.

## Conflicts of interest

The authors declare no conflicts of interest.

## Appendix Supplementary materials

Image, application 1

## Footnotes

Supplementary material associated with this article can be found in the online version at doi:10.1016/j.jped.2024.01.004.
