# Supplementary material for: Exclusive breastfeeding and length of hospital stay in premature infants at a Brazilian reference center for kangaroo mother care
Source: J Pediatr (Rio J). 2024 Mar 21;100(4):392–8. doi: 10.1016/j.jped.2024.01.004 (PMC11331221; doi:10.1016/j.jped.2024.01.004)
Supplement: Supplementary file 1 [file mmc1.docx]

**JPED-D-23-00302**

**SUPPLEMENTARY MATERIAL**


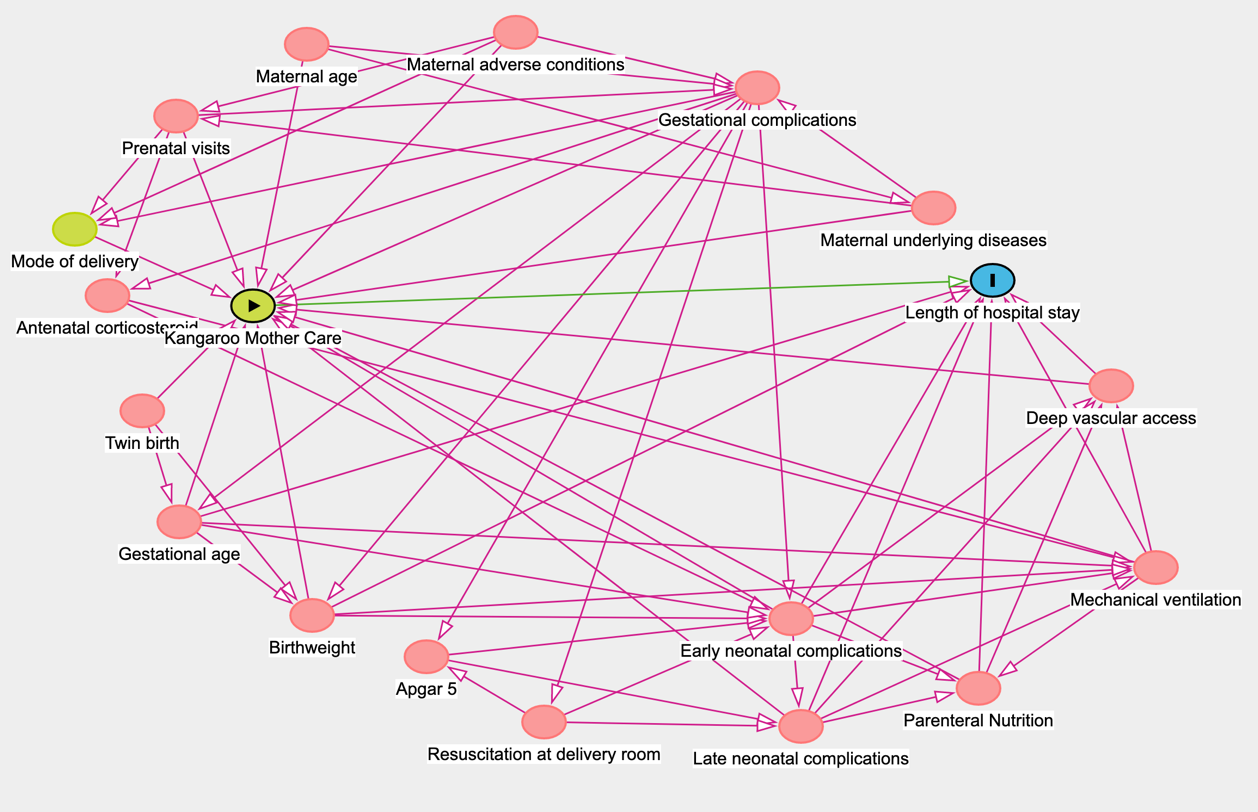


A


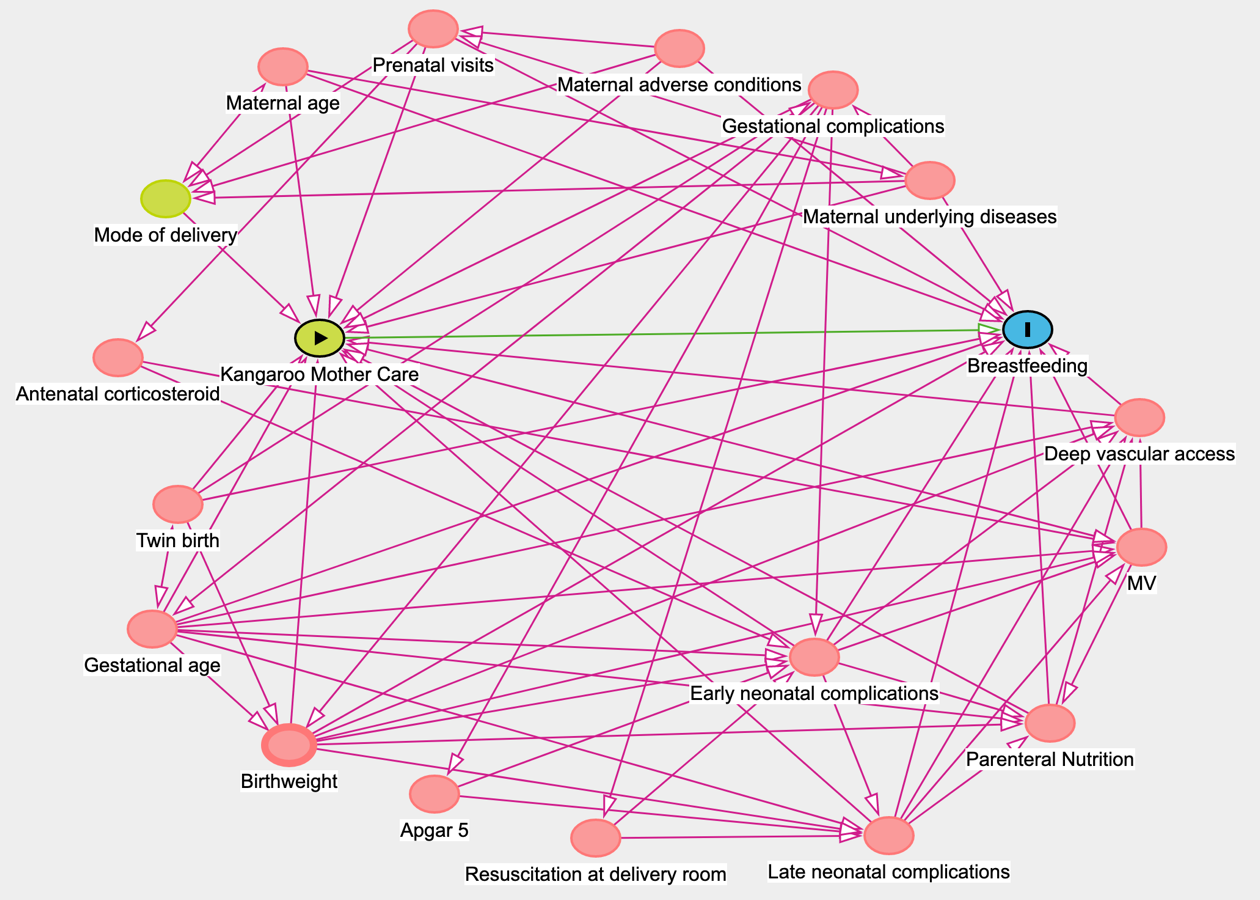


B

**Figure 1** Directed Acyclic Graphics (DAGs) for estimating the effect of Kangaroo care on two outcomes and their respective minimal sufficient adjustment sets in parenthesis: (A) length of hospital stay (gestational age, birth weight, ^1^early and ^2^late neonatal complications, invasive ventilation, parenteral nutrition, deep vascular access); (B) exclusive breastfeeding at discharge (prenatal visits, maternal age, ^3^maternal adverse conditions, ^4^maternal underlying diseases, gestational age, birth weight, early and late and neonatal complications, invasive ventilation, deep vascular access, parenteral nutrition).

^1^ Early neonatal complications (respiratory distress syndrome, pneumonia, pulmonary hemorrhage, early sepsis, persistent ductus arteriosus, shock).

^2^ Late neonatal complications (intraventricular hemorrhage/periventricular leukomalacia, late sepsis, bronchopulmonary dysplasia, necrotizing enterocolitis, retinopathy of prematurity).

^3^ Maternal adverse condition (violence in childhood, domestic violence, illicit drug use, smoking, alcoholism, homeless).

^4^ Maternal underlying diseases (chronic hypertension, hypothyroidism, psychiatric disorders, diabetes, asthma, obesity.
